# Clinically Significant Fatigue: Prevalence and Associated Factors in an International Sample of Adults with Multiple Sclerosis Recruited via the Internet

**DOI:** 10.1371/journal.pone.0115541

**Published:** 2015-02-18

**Authors:** Tracey J. Weiland, George A. Jelinek, Claudia H. Marck, Emily J. Hadgkiss, Dania M. van der Meer, Naresh G. Pereira, Keryn L. Taylor

**Affiliations:** 1 Emergency Practice Innovation Centre, St Vincent’s Hospital, Melbourne, Victoria, Australia; 2 Department of Medicine, The University of Melbourne (St Vincent’s Hospital), Melbourne, Victoria, Australia; 3 Faculty of Medicine, Notre Dame University, Fremantle, Western Australia, Australia; 4 Department of Psychiatry, St Vincent’s Hospital Melbourne, Victoria, Australia; 5 Department of Epidemiology and Preventive Medicine, Monash University, Melbourne, Victoria, Australia; Medical University of Innsbruck, AUSTRIA

## Abstract

**Background:**

Fatigue contributes a significant burden of disease for people with multiple sclerosis (PwMS). Modifiable lifestyle factors have been recognized as having a role in a range of morbidity outcomes in PwMS. There is significant potential to prevent and treat fatigue in PwMS by addressing modifiable risk factors.

**Objectives:**

To explore the associations between clinically significant fatigue and demographic factors, clinical factors (health-related quality of life, disability and relapse rate) and modifiable lifestyle, disease-modifying drugs (DMD) and supplement use in a large international sample of PwMS.

**Methods:**

PwMS were recruited to the study via Web 2.0 platforms and completed a comprehensive survey measuring demographic, lifestyle and clinical characteristics, including health-related quality of life, disability, and relapse rate.

**Results:**

Of 2469 participants with confirmed MS, 2138 (86.6%) completed a validated measure of clinically significant fatigue, the Fatigue Severity Scale. Participants were predominantly female from English speaking countries, with relatively high levels of education, and due to recruitment methods may have been highly pro-active about engaging in lifestyle management and self-help. Approximately two thirds of our sample (1402/2138; 65.6% (95% CI 63.7–67.7)) screened positive for clinically significant fatigue. Bivariate associations were present between clinically significant fatigue and several demographic, clinical, lifestyle, and medication variables. After controlling for level of disability and a range of stable socio-demographic variables, we found increased odds of fatigue associated with obesity, DMD use, poor diet, and reduced odds of fatigue with exercise, fish consumption, moderate alcohol use, and supplementation with vitamin D and flaxseed oil.

**Conclusion:**

This study supports strong and significant associations between clinically significant fatigue and modifiable lifestyle factors. Longitudinal follow-up of this sample may help clarify the contribution of reverse causation to our findings. Further research is required to explore these associations including randomized controlled trials of lifestyle interventions that may alleviate fatigue.

## Introduction

Multiple sclerosis (MS) is a chronic inflammatory disease of the central nervous system (CNS), resulting in neurodegeneration associated with axonal injury and demyelination. The pathogenesis of MS is unclear but is speculated to involve a complex interaction of genetic and environmental determinants[1]. While familial studies suggested a genetic predisposition to MS, evidence of genetic associations in MS clinical course or disease severity is lacking[2]. Data are, however, emerging linking environmental and lifestyle factors to morbidity[3], thereby presenting pivotal opportunities for secondary and tertiary prevention of MS-related fatigue.

The clinical picture of MS is one of heterogeneity, including debilitating symptoms that are both physical and neuropsychiatric in nature [4]. The most common symptom of the illness is fatigue, affecting up to 90% of patients at any one time [5–8]. Among people with MS, fatigue is more severe and disabling than in healthy controls and others with chronic illness [6,9–13]. Approximately two thirds of patients with MS describe fatigue as their most disturbing symptom [5] and fatigue is frequently reported as the first symptom noted by patients with MS prior to diagnosis[14]. Despite the prevalence and potential impact of fatigue in MS, the aetiology of this symptom is poorly understood and treatment options are limited[15,16]. Furthermore, the lack of a unified definition of fatigue may be an impediment to understanding and treating this symptom. Some have described MS-related fatigue as the perception of decreased mental or physical energy that may restrict routines or daily activities.[17]

The consequences of fatigue in the context of MS are myriad; fatigue imposes limitations on socioeconomic status due to its impact on work capacity [18], and is associated with diminished quality of life among people with MS (PwMS) [8]. The experience of fatigue and its effects, however, are potentially intertwined. Fatigue in MS is well known to be associated with depression but is itself a symptom of depression. It is not surprising to note then that depression, and several other MS-related symptoms frequently cluster together with fatigue. Among these are emotional distress, depression, disability [19], cognitive dysfunction[20] and heat sensitivity [21], each of which has been shown to exacerbate perceptions of fatigue.

Given the overlap in symptoms that cluster with fatigue, and the strong interplay between them, there are obvious complexities in determining an underlying pathophysiological mechanism contributing to fatigue symptoms. “Primary fatigue”, which is directly attributable to MS disease mechanisms, may result from inflammatory processes associated with immune activation, demyelination, axonal loss, or neuroendocrine disturbance [16]. These factors, however, account for only a small amount of variance in fatigue among those with MS[15,16], leading some to speculate that other factors may affect the perception of fatigue. In this regard, “secondary fatigue”, loosely defined as fatigue indirectly caused by the pathological consequences of MS, includes that caused by MS-related sleep disturbance, depression and inactivity[16], disability status, MS subtype, and iatrogenic mechanisms [22], and catastrophic inferential thinking regarding fatigue symptoms [23].

Since fatigue in MS is so poorly understood yet contributes a significant burden of the disease, identifying variables that affect clinically significant fatigue is important, and may provide methods of minimising this debilitating symptom in PwMS. This study is part of a wider research project, the Health Outcomes and Lifestyle Interventions in a Sample of People with Multiple Sclerosis (the HOLISM study). Our aim with this component of the research was to explore the association between clinically significant fatigue and a wide range of demographic factors, morbidity outcomes (health-related quality of life (HRQOL), disability and relapse rate) and lifestyle factors (diet, smoking, alcohol use, exercise, meditation), and use of medications and supplements in a large international sample of PwMS.

## Method

### Participants and recruitment

The study methodology and participant demographics for the HOLISM study have been described previously[24]. To summarise, participants were recruited over 15 weeks via Web 2.0 platforms, including social media, through which an online survey was distributed. Facebook groups and pages designed for people with MS and having over 500 followers were targeted as potential recruitment sites. On a weekly or bi-weekly basis postings on these websites were made with links to the survey. Additionally, subscribers to the OMS website (http://www.overcomingmultiplesclerosis.org) were invited by email to participate with several reminders over the recruitment period.

The web-based tool, SurveyMonkey, was used to present participants with a participant information sheet, an electronic consent indicator, and the survey itself. Those eligible for the study included adults self-reporting a formal diagnosis of MS by a medical doctor. Respondents were excluded if they were under 18 years of age.

### Ethics statement

Ethics approval was granted by St Vincent’s Hospital Melbourne Human Research Ethics Committee (LRR 055/12). This included approval for the method of consent used.

### Data collection and tools used

The survey was comprehensive, consisting of 163 questions in total, and took approximately 40 minutes to complete, with the ability to suspend and re-enter the survey if required. Where possible the survey used validated tools that had sound psychometric properties and had been tested in a similar population. The survey collected contact information, demographic data and self-reported data for disease profile, medications and supplements, and lifestyle factors.

**Demographic data**. Demographic items assessed age, gender, years since MS diagnosis, age at diagnosis, marital status, number of children, and employment status. We also collected data on country of birth which was subsequently collapsed into six groups (representing the five modal countries and other) for measures of association. Data for location (country and city) were also collected permitting derivation of latitudinal data.

**Social support**. The Single Item Measure of Social Support (SIMSS)[25] was used to determine number of close relationships. Response options were: none, 1 person, 2–5 people, 6–9 people, or 10 or more people. The latter two categories were collapsed together for analyses.

**Disease profile**. Indicators of disease profile included clinically significant fatigue, type of MS, depression risk, level of disability, HRQOL, number of comorbidities, and for those with relapsing-remitting MS, relapse rate and disease activity.

Data for clinically significant fatigue were collected using the Fatigue Severity Scale (FSS). This is a widely validated tool consisting of nine fatigue-related statements rated on a seven-point scale (disagree to agree)[11]. It has good internal consistency, stability, and sensitivity to change over time and has been used in several MS populations internationally[26,27]. A mean score greater or equal to 4 has been used by others as the cut off indicating clinically significant fatigue[27–29], and was adopted as the cutoff in the present study. To derive this summary score, full item completion was required.

Depression risk, or strong likelihood of depression, was assessed using the patient health questionnaire (PHQ-2), a short form of the PHQ-9 depression screening tool which has been validated in 173 patients with MS[30]. The PHQ-2 had good construct validity in a sample of 6000 patients recruited from primary care settings and obstetric and gynaecology specialist clinics[31]. In a sub-sample of 580 of these patients that were further interviewed by a mental health professional the PHQ-2 showed good criterion validity, and 83% sensitivity and 92% specificity for major depression for scores equal to or great than 3[31].

Health-related quality of life was assessed using the Multiple Sclerosis Quality Of Life-54 (MSQOL-54) scale, a measure of HRQOL developed from the RAND 36-Item Health Survey (SF-36) and supplemented with 18 additional items. It comprises 52 items distributed across 12 scales, giving rise to physical and mental health composites, and two single items, and has been extensively validated[32].

Level of disability was assessed using the Patient-Determined Disease Steps (PDDS)[33], a self-reported tool which can be used as a surrogate tool for the Expanded Disability Status Scale (EDSS) commonly used to assess gait disability. It is scored on an ordinal scale from 0 (normal) to 8 (bed bound) and correlates well with the EDSS (Spearman rank r = 0.64) and the Multiple Sclerosis Functional Composite (Spearman Rank r = 0.58) and has excellent concordance between raters (kappa 0.8). The PDDS has been used in a several studies of PwMS. For analyses the PDDS was collapsed from nine to three categories (normal, mild disability, moderate disability = “normal/some disability”; gait disturbance, cane, late cane = “gait/cane disability”; bilateral support, wheelchair, and bedridden = “major mobility support”).

The Self-Administered Comorbidity Questionnaire (SCQ) was used to assess co-morbidities. This tool determines if the co-morbidity limits activities and whether treatment is currently being received. It has previously been used in a study of people with MS [34]. In our study, two arthritic co-morbidities were combined into one. All listed conditions were summed to determine an estimate of the number of co-morbidities each participant reported. Participants were then categorized as having: ‘none’, ‘1’, ‘2’, ‘3’, ‘4’, or ‘5 or more’, co-morbidities.

For those with relapsing remitting MS, we explored the number of doctor-diagnosed relapses over the previous 12 months and the preceding five years, as reported by the participant. Five year annualised relapse rates were calculated by dividing the number of relapses over five years by the number of years of disease with an upper limit of five. We then derived the pre-determined variable “disease activity”: where specialist-determined relapse rate in the preceding 12 months exceeded the 5 year annualised relapse rate, disease activity was categorised as increasing; where relapse rate for the preceding 12 month was lower than the five year annualised rate, disease activity was categorised as decreasing; and where 12 month relapse rate was the same as the 5 year annualised rate, disease activity was categorised as stable.

**Lifestyle factors**. Alcohol use and smoking status and level were assessed using a researcher devised tool with alcohol use categorized as described previously[35]. Dietary habits were assessed using the dietary habits questionnaire (modified DHQ)[36]. Items assessing salt use were excluded for ease of scoring (resulting in a DHQ maximal total score of 100) and lack of relevance of salt intake to MS outcome measures. DHQ items related to alcohol intake were also excluded as this was examined in more detail in other parts of the survey. The remaining 20 items were scored from 1–5, giving rise to a summary score with a possible range of 20–100, with higher scores indicating more healthy dietary habits. Data were categorized into quartiles for inferential analyses.

Data were also collected for meditation frequency (‘never’, ‘less than once per week’ or ‘once or more per week’). Physical activity was assessed using the International Physical Activity Questionnaire (IPAQ), which assesses the duration and frequency of vigorous and moderate physical activity, walking and sitting over the last 7 days. The IPAQ has been validated in MS populations[37,38]. Participants were categorized according to scoring instructions as low active, moderate active, or high active.

Body mass index (BMI) was calculated and categorized according to World Health Organization standards[39].

**Medications and supplements**. Participants were asked about the specific medications taken currently and previously using a researcher-generated list of first and second generation disease-modifying drugs (DMDs) and common MS drugs. For the purpose of analysis, participants were categorized according to whether they took a DMD currently only, currently and previously, previously only, or never. Additionally, to facilitate exploratory analyses for each of the four DMDs used most commonly by this sample (interferons, glatiramer acetate, fingolimod, and natalizumab), we collapsed data based on responses to all medications taken to form new variables: “takes the DMD under investigation (one of either interferons, glatiramer acetate, fingolimod, or natalizumab)” “takes no DMDs” and “takes a different DMD” (other than that used in the analysis).

Respondents self-reported current vitamin D supplementation from which an average daily dosage was calculated. Participants were grouped as ‘none’, ‘1–5000 international units’ (IU) or >5000 IU.

Omega-3 fatty acid supplementation used in the last 12 months was analysed in terms of omega-3 supplementation taken (yes/no), average daily dose (mls), and type: ‘flaxseed oil’, ‘fish oil and high strength fish oil’, ‘both’ or ‘none’.

### Data analysis

Data were analysed using IBM SPSS Statistics 22.0 (IBM Corporation). We undertook bivariate and multivariate analyses. Continuous data are summarised using mean (95% CI) and categorical data using number and percentage. Comparisons between two groups on continuous endpoints were undertaken using independent samples t-test, comparisons involving three or more independent groups were undertaken using analysis of variance (ANOVA) with Least Significant Difference as post-hoc analyses. For categorical data involving two by two contingency tables, data were analysed using Fisher’s Exact Test and for categorical data involving more than two groups, Pearson’s Chi Square was used with adjusted standardised residuals used to indicate under- or over-representation of groups.

Bivariate analyses were used to explore the relationship between socio-demographic factors, disease-specific variables, and clinically significant fatigue to determine factors that should be included as covariates in regression modeling. Binary logistic regression was used to predict clinically significant fatigue (those with a FSS mean score ≥4). Relatively stable factors including demographic (gender, age, education status, employment status, marital status, number of children, years since diagnosis) and disease profile factors (number of comorbidities, type of MS, level of disability, HRQOL, relapse rate, and disease activity), were assessed in a series of separate regression models that included only the independent variable of interest. This approach was selected due to substantial redundancies in multivariable models that contributed to multi-collinearity thereby violating this assumption of regression.

In a second series of individual regression models we assessed modifiable factors such as lifestyle factors (smoking status, alcohol use, exercise, meditation, social support, BMI, dietary habits including fish consumption), and medication or supplement use (DMD use, vitamin D, omega 3, omega 3 type) and report the crude odds ratios and adjusted odds ratios (adjusted for age, gender, level of education, employment status, marital status, number of children, number of comorbidities, level of disability). Since we have previously demonstrated a strong association between “clinically significant fatigue” and positive screen for “depression risk” (OR 9.06 (95% CI 6.14–13.38)) [40], we also included this as a covariate. No adjustments were made for “type of MS” and “time since diagnosis” since these variables were highly correlated with “age” and “level of disability” respectively. No adjustment was made for “age at diagnosis” as this was highly correlated with “current age”, and both “relapse rate” and “disease activity” were excluded as covariates as these data were provided for those with relapsing remitting MS only. “HRQOL” was not included as a covariate as these data were significantly associated with “comorbidities”, “depression risk”, and “level of disability”.

Preliminary tests of the assumptions of logistic regression were performed, including an examination of multi-collinearity to ensure that continuous independent variables were not closely correlated (having a bivariate correlation >0.70). Odd ratios and 95% CIs are reported.

For all inferential tests, two-tailed tests of significance were used and the criterion for significance was set at. 05. All percentages reported have been adjusted for missing data (due to item non-completion) on an item by item basis.

## Results

### Participation, demographics, and fatigue

Of 2469 respondents with a confirmed diagnosis of MS, 2138 (86.6%) answered questions on the FSS allowing a mean score to be calculated and subsequent categorisation as clinically significantly fatigued or not clinically significantly fatigued.

The full sample demographics have been documented previously[24]. Among respondents completing the FSS (n = 2138), there was a preponderance of women (n = 1743, 82.3%) and the mean age was 45.5 years. Average age at diagnosis was 38.0 years, and mean time since diagnosis was 8.5 years. The majority were married (1303, 61.8%) or cohabiting (277, 13.1%). Approximately one third of participants were located in the United States of America, approximately one quarter in Australia, and around one sixth in the United Kingdom. The remaining respondents to the FSS questions resided in 50 other countries or territories. Respondents to the FSS questions represented 72 countries of birth.

As previously reported, respondents had a median overall score on the FSS of 4.9 (IQR 3.2–6.1)[24]. Approximately two thirds of our sample (1402/2138; 65.6% (95% CI 63.7–67.7)) were scored as having clinically significant fatigue on the FSS.

There were no significant differences between those who completed the FSS and the 13.4% (n = 331) of our sample that did not, in terms of age, gender, number of children, type of MS, BMI, level of disability, positive screen for depression, and number of comorbidities (data not shown). Non-completion was significantly more likely among respondents whose country of birth was USA (121/772, 15.7%), or a country other than the five modal countries of birth (79/417, 18.9%), p<.001; if their marital status was single (63/359, 17.5%), or separated/divorced (47/257, 18.3%), p = .001; and if they were unemployed (35/196, 17.9%) or retired due to disability (91/570, 16.0%), p = .009.

### Stable factors associated with fatigue: Socio-demographic and clinical status factors

Numerous demographic factors were associated with clinically significant fatigue. Among them were older age, female gender, greater number of years since diagnosis, being separated, divorced or widowed, having several children, a lower level of education, being retired due to disability, and having USA or UK as country of birth (Table 1). Latitude at the time of survey completion was not associated with fatigue (OR 1.027 (95% CI 0.99–1.065), p = .140).

**Table 1:** Socio-demographic factors associated with clinically significant fatigue.

Outcome	Subgroup	No Clinically Significant Fatigue (FSS mean score <4)	Clinically Significant Fatigue (FSS mean score ≥4)	P	Crude OR (95%CI)
Age, mean		44.1 (43.3–44.9)	46.3 (45.8–46.9)	<.001	1.02 (1.01–1.03)***
	Total N	719 (34.6)	1362 (65.4)		
Gender	Male	147 (39.2)	228 (60.8)	.036	0.78 (0.62–0.98)* reference
	Female	582 (33.4)	1161 (66.6)		
	Total N	729 (34.4)	1389 (65.6)		
Years Since diagnosis, mean		7.6 (7.10–8.10)	9.0 (8.65–9.42)	<.001	1.03 (1.02–1.04) ***
	Total N	733 (34.6)	1397 (65.4)		
Age at diagnosis, mean		37.4 (36.7–38.2)	38.3 (37.7–38.8)	.075	1.01 (.99–1.02)
	Total N	716 (34.6)	1356 (65.6)		
Marital status	Married, cohabiting, partnered	553 (35.0)	1027 (65.0)	<.001	reference
	Single	121 (40.9)^††^	175 (59.1)^†^		0.78 (.604–1.01)
	Separated/Divorced	49 (23.3) ^†^	161 (76.7) ^††^		1.77 (1.26–2.48) ***
	Widowed	4 (16.7)	20 (83.3)		2.692 (0.916–7.916)
	Total N	727 (34.5)	1383 (65.5)		
Children	0	278 (41.7)^††^	388 (58.3) ^†^	<.001	reference
	1	108 (33.3)	216 (66.7)		1.43 (1.09–1.89) *
	2	213 (32.2)	448 (67.8)		1.51 (1.20–1.89) ***
	3+	123 (26.7) ^†^	337 (73.3) ^††^		1.96 (1.52–2.54) ***
	Total N	722 (34.2)	1389 (65.8)		
Employment Status	Work Full time	333 (47.0) ^††^	376 (53.0) ^†^	<.001	reference
	Work Part time	161 (35.9)	288 (64.1)		1.58 (1.24–2.02) ***
	Stay at home parent/carer	53 (31.4)	116 (68.6)		1.94 (1.36–2.77) ***
	Unemployed	47 (29.2)	114 (70.8)		2.15 (1.48–3.11) ***
	Retired due to disability	66 (13.8) ^†^	413 (86.2) ^††^		5.54 (4.11–7.47) ***
	Retired due to age	24 (35.8)	43 (64.2)		1.59 (0.94–2.67)
	Other (includes student)	51 (51.5) ^††^	48 (48.5) ^†^		0.834 (0.55–1.27)
	Total N	735 (34.5)	1398 (65.5)		
Education status	Secondary school or lower	118 (22.9) ^†^	398 (77.1) ^††^	<.001	2.63 (2.01–3.45) ***
	Vocational training	101 (30.1)	234 (69.9)		1.81 (1.35–2.42) ***
	Bachelor’s degree	295 (38.0) ^††^	481 (62.0) ^†^		1.27 (1.01–1.60) *
	Postgraduate degree	220 (43.8) ^††^	282 (56.2) ^†^		Reference
	Total N	734 (34.5)	1395 (65.5)		
Country of Birth	Australia	172 (37.6)	285 (62.4)	.005	1.14 (0.85–1.51)
	Canada	33 (35.5)	60 (64.5)		1.25 (0.77–2.00)
	New Zealand	56 (36.1)	99 (63.9)		1.21 (0.82–1.79)
	United Kingdom	149 (33.7)	293 (66.3)		1.35 (.00–1.81) *
	USA	189 (29.0) ^†^	462 (71.0) ^††^		1.67 (1.27–2.21) ***
	Other	137 (40.7) ^††^	200 (59.3) ^†^		Reference
	Total N	736 (34.5)	1399 (65.5)		

Data are number (%) unless otherwise specified.

^††^ denotes category significantly over-represented according to adjusted standardised residuals.

^†^ Denotes category significantly under-represented according to adjusted standardised residuals.

*<0.05

***≤0.001

Those with benign MS had a greater likelihood of screening negative for clinically significant fatigue compared to those with a relapsing remitting type of MS (Table 2). By contrast those with progressive relapsing type of MS had more than a four-fold increase in odds of fatigue compared with people with relapsing remitting type of MS. This effect however was observed for a relatively small sample of people with progressive relapsing type of MS thereby giving rise to a wide 95% CI (Table 2). Respondents with primary or secondary progressive illness had approximately two and half time the odds of severe fatigue compared to those with relapsing remitting type (Table 2).

**Table 2:** Bivariate associations between clinically significant fatigue and clinical factors.

Outcome	Subgroup	No Clinically Significant Fatigue (FSS mean score <4)	Clinically Significant Fatigue (FSS mean score ≥4)	P	Crude OR (95%CI)
Type of MS	Relapsing-remitting	502 (38.3)^††^	808 (61.7) ^†^	<.001	Reference
	Benign	54 (58.1) ^††^	39 (41.9) ^†^		0.45 (0.24–0.69)***
	Primary progressive	28 (17.9) ^†^	128 (82.1) ^††^		2.84 (1.86–4.34)***
	Secondary progressive	49 (20.4) ^†^	191 (79.6) ^††^		2.42 (1.75–3.38)***
	Progressive relapsing	6 (13.3) ^†^	39 (86.7) ^††^		4.04 (1.70–9.61)***
	Other/Unsure	93 (32.5)	193 (67.5)		1.29 (.98–1.69)
	Total N	732 (34.4)	1398 (65.6)		
Level of disability (PDDS)	No, mild, moderate disability	583 (49.4) ^††^	598 (50.6) ^†^	<.001	Reference
	Gait or cane disability	105 (14.3) ^†^	631 (85.7) ^††^		5.86 (4.63–7.42)***
	Major mobility support	46 (21.4) ^†^	169 (78.6) ^††^		3.58 (2.55–5.06)***
	Total N	734 (34.4)	1398 (65.6)		
HRQOL, mean (MSQOL-54)	Overall HQOL domain	78.2 (77.10–79.23)	61.5 (61.5–62.48)	<.001	0.94 (0.93–0.94)***
	Total N	720 (34.4)	1370 (65.6)		
	Physical health composite	77.8 (76.6–79.0)	49.6 (48.6–50.6)	<.001	0.91 (0.90–0.92)***
	Total N	623 (34.6)	1179 (65.4)		
	Mental health composite	80.1 (79.02–81.22)	60.3 (59.14–61.37)	<.001	0.94 (0.93–0.94)***
	Total N	709 (34.6)	1338 (65.4)		
	Energy Domain	62.3 (61.0–63.5)	33.7 (32.7–34.6)	<.001	0.92 (0.92–0.93)***
	Total N	735 (34.5)	1397 (65.5)		
Comorbidities (SCQ), number	0	352 (49.5) ^††^	359 (50.5) ^†^	<.001	Reference
	1	206 (36.7)	355 (63.3)		1.69 (1.35–2.12)***
	2	113 (26.2) ^†^	318 (73.8) ^††^		2.76 (2.13–3.58)***
	3	40 (16.1) ^†^	209 (83.9) ^††^		5.12 (3.54–7.41)***
	4	16 (15.4) ^†^	88 (84.6) ^††^		5.39 (3.10–9.37)***
	5+	7 (9.0) ^†^	71 (91.0) ^††^		9.95 (4.51–21.92)***
	Total N	734 (34.4)	1400 (65.6)		
Relapse rate, mean (95% CI)	12-month doctor diagnosed relapse rate	0.51 (0.44–0.58)	0.84 (0.75–0.92)	<.001	1.43 (1.25–1.64)***
	Total N	497 (38.8)	784 (61.2)		
Disease activity	Decreasing	220 (42.1) ^††^	302 (57.9) ^†^	<.001	Reference
	Same	159 (47.6) ^††^	175 (52.4) ^†^		0.80 (0.61–1.06)
	Increasing	96 (26.3) ^†^	269 (73.7) ^††^		2.04 (1.53–2.73)***
	Total N	475 (38.9)	746 (61.1)		

Data are number (%) unless specified.

^*γ*^relapsing-remitting only

^††^ denotes category significantly over-represented according to adjusted standardized residuals.

† Denotes category significantly under-represented according to adjusted standardized residuals.

***≤0.001

Morbidity-related factors associated with clinically significant fatigue were having gait or cane disability or requiring major mobility support (PDDS), having a lower overall HRQOL (MSQOL-54), and lower scores on the physical health composite, mental health composite, and energy subscore (Table 2). For every increase of one point on overall HRQOL (MSQOL-54), the odds of clinically significant fatigue reduced by 0.06 (OR. 94 (95% CI. 93-.94). People with five or more comorbidities had over nine times the odds of fatigue compared to those with no comorbidities (Table 2). Among respondents with relapsing remitting MS, having a higher median relapse rate, and having increasing disease activity were associated with a greater likelihood of clinically significant fatigue.

### Modifiable factors associated with clinically significant fatigue

Lifestyle factors associated with clinically significant fatigue included being a current smoker, having a low consumption of alcohol, having a low level of physical activity, having a lower score on the dietary habits questionnaire, and having a low level of fish consumption (<once/week), having fewer than 6 close relationships, and being overweight or obese (Table 3). By contrast, consuming fish three or more times a week, having never smoked, consuming alcohol moderately, and engaging in physical activity that was moderate or high was associated with a significantly lower likelihood of fatigue. Meditation was not significantly associated with changes in fatigue. Taking vitamin D, flaxseed oil, or 11–20 mls of omega 3 supplement reduced the (unadjusted) odds of fatigue by more than half. Being overweight or obese increased the (unadjusted) odds of fatigue by 1.7 and 2.9 times, respectively. Taking a DMD currently or previously, and taking a DMD previously only were each associated with a 1.5 times increase in the (unadjusted) odds of fatigue when compared to those that had never taken a DMD.

**Table 3:** Crude and adjusted odds ratios (95% confidence intervals), for modifiable factors predicting clinically significant fatigue.

Outcome	Category	No Clinically Significant Fatigue, n(%).(FSS mean score <4)	Clinically Significant Fatigue, n(%).(FSS mean score ≥4)	p	Crude OR (95% CI)	Adjusted OR (95% CI)
Smoking	Never	381 (37.3) ^††^	640 (62.7) ^†^	<0.001	1.00 (reference)	1.00 (reference)
	Former	307 (35.6)	556 (64.4)		1.08 (0.89–1.30)	0.79 (0.63–1.00) *
	Current	47 (19.0) ^†^	201 (81.0) ^††^		2.55 (1.81–3.58)***	1.34 (0.87–2.08)
	Total N	735 (34.5)	1395 (65.5)			
Alcohol	Low	378 (29.2) ^†^	916 (70.8) ^††^	<0.001	1.00 (reference)	1.00 (reference)
	Moderate	345 (42.7) ^††^	463 (57.3) ^†^		0.55 (0.46–0.67) ***	0.75 (0.61–0.94) *
	High	6 (35.3)	11 (64.7)		0.76 (0.28–2.06)	0.70 (0.23–2.14)
	Total N	729 (34.4)	1390 (65.6)			
Exercise (IPAQ)	Low	169 (19.4) ^†^	702 (80.6) ^††^	<0.001	1.00 (reference)	1.00 (reference)
	Moderate	262 (38.8) ^††^	413 (61.2) ^†^		0.38 (0.30–0.48) ***	0.52 (0.40–0.67) ***
	High	297 (52.3) ^††^	271 (47.7) ^†^		0.22 (0.17–0.28) ***	0.32 (0.24–0.42) ***
	Total N	728 (34.4)	1386 (65.6)			
Vitamin D	None	87 (23.1) ^†^	289 (76.9) ^††^	<0.001	1.00 (reference)	1.00 (reference)
	1–5000 IU	448 (35.1)	829 (64.9)		0.56(0.43–0.73) ***	0.70 (0.51–0.95) *
	> 5000 IU	175 (41.8) ^††^	244 (58.2) ^†^		0.42 (0.31–0.57) ***	0.59(0.41–0.85) **
	Total N	710 (34.3)	1362 (65.7)			
Omega 3	No	217 (28.4) ^†^	548 (71.6) ^††^	<0.001	1.00 (reference)	1.00 (reference)
	Yes	514 (37.8) ^††^	845 (62.2) ^†^		0.65 (0.54–0.79) ***	0.86 (0.68–1.08)
	Total N	731 (34.4)	1393 (65.6)			
Omega 3 type	None	217 (28.4) ^†^	548 (71.6) ^††^	<0.001	1.00 (reference)	1.00 (reference)
	Fish oil^	275 (37.0)	469 (63.0)		0.68 (0.54–0.84) ***	0.84 (0.65–1.08)
	Flaxseed oil	89 (46.6) ^††^	102 (53.4) ^†^		0.45 (0.33–0.63) ***	0.63 (0.43–0.93) *
	Both fish and flaxseed oil	143 (38.2)	231 (61.8)		0.64 (0.49–0.83) **	0.97 (0.71–1.33)
	Total N	724 (34.9)	1350 (65.1)			
Omega 3 dose	None	217 (28.4) ^†^	548 (71.6) ^††^	<.001	1.00 (reference)	1.00 (reference)
	1–10mls	339 (36.1)	599 (63.9)		0.70 (0.57–0.86) ***	0.90 (0.70–1.14)
	11–20mls	101 (46.5) ^††^	116 (53.5) ^†^		0.46 (.33–0.62) ***	0.72 (0.49–1.03)
	21+mls	56 (40.6)	82 (59.4)		0.58 (.40-.84) **	0.76 (0.49–1.17)
	Total N	713 (34.6)	1345 (65.4)			
Fish consumption	Less than once per week	160 (26.1) ^†^	454 (73.9) ^††^	<0.001	1.00 (reference)	1.00 (reference)
	1–2 times per week	280 (32.9)	571 (67.1)		0.72 (0.57–0.91) **	0.88(0.67–1.16)
	3 or more days per week	296 (44.4) ^††^	371 (55.6) ^†^		0.44 (0.35–0.56) ***	0.66 (0.49–0.89) **
	Total N	736 (34.5)	1396 (65.5)			
Meditation	Never	337 (33.2)	678 (66.8)	.363	1.00 (reference)	1.00 (reference)
	Less than once per week	165 (34.5)	313 (66.5)		0.94 (0.75–1.19)	1.11 (0.85–1.46)
	Once or more per week	234 (36.6)	405 (63.4)		0.86 (0.70–1.06)	1.02 (0.79–1.30)
	Total N	736 (34.5)	1396 (65.5)			
Social support	6 or more	122 (42.8) ^††^	163 (57.2) ^†^	0.001	1.00 (reference)	1.00 (reference)
	2–5	439 (28.6)	823 (71.4)		1.40 (1.08–1.82) *	1.16 (0.86–1.58)
	1	132 (34.8) ^†^	329 (65.2) ^††^		1.87 (1.37–2.54) ***	1.30 (0.90–1.87)
	None	33 (30.8)	74 (69.2)		1.68 (1.05–2.69) *	0.94 (.53–1.68)
	Total N	726 (34.3)	1389 (65.7)			
Body Mass Index (WHO classification)	Normal	**473** (41.8)	659 (58.2)	<0.001	1.00 (reference)	1.00 (reference)
	Underweight	34 **(39.8)** ^††^	53(60.2) ^†^		1.09 (0.70–1.69)	0.93 (0.56–1.55)
	Overweight	143 (29.2) ^†^	347 (70.8) ^††^		1.74(1.39–2.19) ***	1.46 (1.12–1.90) **
	Obese	81 (20.0) ^†^	325 (80.0) ^††^		2.88 (2.20–3.78) ***	1.75 (1.28–2.40) ***
	Total N	732 (34.6)	1384 (65.4)			
Dietary Habits Questionnaire (modified)	Upper (fourth) quartile	246 (47.0) ^††^	277 (53.0) ^†^	<0.001	1.00 (reference)	1.00 (reference)
	Third quartile	180 (37.9)	295 (62.1)		1.46 (1.13–1.87) **	1.31 (0.98–1.75)
	Second quartile	150 (31.3)	329 (68.7)		1.95 (1.50–2.52) ***	1.52 (1.12–2.07) **
	Lower (first) quartile	104 (21.5) ^†^	379 (78.5) ^††^		3.24 (2.45–4.27) ***	2.02 (1.44–2.83) ***
	Total N	680 (34.7)	1280 (65.3)			
Disease Modifying Drug use	Never	278 (39.7) ^††^	423 (60.3) ^†^	<.001	1.00 (reference)	1.00 (reference)
	Current only	245 (35.7)	441 (64.3)		1.183 (0.95–1.47)	1.37 (1.05–1.79)**
	Current and previous	108 (27.6) ^†^	284 (72.4) ^††^		1.73 (1.32–2.26) ***	1.76 (1.26–2.44) ***
	Previous but not current	104 (29.1) ^†^	254 (70.9)^††^		1.61 (1.22–2.11) ***	1.36 (0.97–1.90)
	Total N	735 (34.4)	1402 (65.6)			

Adjusted OR: Odds ratios of screening positive for fatigue adjusted for: age, gender, level of education, employment status, marital status, country of birth, number of children, number of comorbidities, level of disability, and positive depression screen. Neither MS type nor years since diagnosis were included as covariates since these variables were highly correlated with disability and age respectively. Age at diagnosis was not included as a covariate as it was highly correlated with current age, and both relapse rate and disease activity were excluded as covariates as these data were provided for those with relapsing remitting MS only. HRQOL was not included as a covariate as these data were significantly associated with comorbidities, depression, and disability.

^††^ denotes category significantly over-represented according to adjusted standardized residuals.

† Denotes category significantly under-represented according to adjusted standardized residuals.

*<0.05

**<0.01

***≤0.001

**Predictors of fatigue controlling for stable factors**. After adjusting for age, gender, level of education, employment status, marital status, country of birth, number of children, number of comorbidities, level of disability, and positive depression screen, several modifiable variables reduced the odds for clinically significant fatigue: consuming moderate quantities of alcohol, consuming fish three or more times a week, supplementation with vitamin D, and taking flaxseed oil (compared with no omega 3 supplement) significantly reduced the adjusted odds of fatigue (Table 3). Having a normal BMI reduced the adjusted odds of fatigue compared to being obese (OR 0.57 (95% CI 0.42–0.78)), or overweight (OR 0.68 (95% CI 0.52–0.89)). Having never used a DMD offered significantly reduced odds compared to current only (OR 0.72 (95% CI 0.56–0.95) and current and previous DMD use (OR 0.57 (95% CI 0.41–0.79) (Table 3). Having a healthy diet (modified DHQ score in 4^th^ quartile) reduced the adjusted odds of fatigue by half compared to a poor diet (modified DHQ score in 1^st^ quartile). The greatest benefit however was observed for high levels of physical activity; those who exercised vigorously three times a week or undertook mild, moderate or vigorous exercise over seven days were almost three times less likely to screen positive for fatigue after adjusting for covariates (Table 3). Meditation, social support and current smoking were not associated with clinically significant fatigue after adjusting for stable factors.

For respondents taking natalizumab alone or interferon alone compared to another single DMD or no DMD there was no significant increase in likelihood of clinically significant fatigue (Table 4). However, compared with other groups, a significantly higher proportion of those taking fingolimod screened positive for clinically significant fatigue. Those taking a single DMD other than glatiramer acetate were significantly more likely to screen positive for fatigue. When all four medications were compared against one another and no DMD use, those taking fingolimod were significantly more likely to have clinically significant fatigue. Among participants with “gait or cane” or “major disability” (PDDS), those taking fingolimod, natalizumab, glatiramer acetate or interferons, were not significantly more likely to have clinically significant fatigue when compared with those not taking these medications (data not shown).

**Table 4:** Association between specific medication use and clinically significant fatigue.

Medication Subgroup	**No Clinically Significant Fatigue** (FSS mean score <4)	**Clinically Significant Fatigue** (FSS mean score ≥4)	P
Interferon only	120 (30.8)	269 (69.2)	.169
another single DMD	230 (33.9)	448 (66.1)	
No DMD	382 (36.1)	677 (63.9)	
Total N	732 (34.4)	1394 (65.6)	
Natalizumab only	37 (31.1)	82 (68.9)	.271
Another single DMD	315 (33.1)	637 (66.9)	
No DMD	382 (36.1)	677 (63.9)	
Total N	734 (34.5)	1396 (65.5)	
Fingolimod only	18 (22.8) ^†^	61 (77.2) ^††^	.039
Another single DMD	333 (33.4)	664 (66.6)	
No DMD	382 (36.1)	677 (63.9)	
Total N	733 (34.3)	1402 (65.7)	
Glatiramer acetate only	166 (38.0)	271 (62.0)	.003
Another single DMD	184 (29.2) ^†^	446 (70.8) ^††^	
No DMD	382 (36.1)	677 (63.9)	
Total N	732 (34.4)	1394 (65.6)	
Takes natalizumab only	37 (31.1)	82 (68.9)	.025
Takes fingolimod only	18 (22.8) ^†^	61 (77.2) ^††^	
Takes glatiramer acetate only	166 (38.0)	271 (62.0)	
Takes interferons only	120 (30.8)	269 (69.2)	
Takes no DMD	382 (36.1)	677 (63.9)	
Total N	723 (34.7)	1360 (65.3)	

Data are number (%).

^††^ denotes category significantly over-represented according to adjusted standardized residuals.

^†^ denotes category significantly under-represented according to adjusted standardized residuals.

## Discussion

This study provides a unique, preliminary analysis of the associations between multiple demographic, clinical and modifiable lifestyle variables and clinically significant fatigue in a large international sample of adults with MS. Clinically significant fatigue in this sample was high, with 65.6% screening positive using the FSS. With the exception of the landmark NARCOMS study, which reported the prevalence of clinically significant fatigue to be 74% [41], this figure concurs strongly with previous reports [10,14,42,43]. The high prevalence of fatigue in our sample is particularly noteworthy since those in our sample may have a greater likelihood than others of adopting health-seeking behaviours, as suggested by their higher educational attainment, use of internet resources, and previous reports regarding diet and lifestyle [24,44]. However, since the sample comprised a greater proportion of females to males (4.7:1, F:M) than the estimated incidence ratio of 3.2:1[45], and, contrary to findings by others [41], females had higher levels of fatigue than males, it is possible that the effects of gender influenced the fatigue levels reported for this sample overall.

The mechanisms underpinning fatigue in MS are poorly understood and are likely to be multiple. Fatigue is considered a primary symptom in MS and is associated, in part, with a range of underlying pathophysiological processes involving the immune system and accompanying CNS damage [22]. Included among these are demyelination, axonal loss, neuroendocrine dysregulation[15], microstructural abnormalities and regional atrophy [46], altered patterns of cerebral activation[22], inflammation and accompanying immunoactivation; pro-inflammatory cytokines (particularly TNF alpha, IL-6, and interferon-γ) are also thought to contribute to MS-related fatigue[47–49]. While these factors are pivotal in the genesis of primary fatigue, it is pertinent to note that a range of factors may contribute to secondary fatigue that develops consequent to disease burden.

The socio-economic consequences of fatigue in PwMS are significant. Fatigue is associated with loss of or reduced hours of employment, lower quality of life[14,42] and reduced ability to carry out day to day tasks. This, and the fact that up to 90% of PwMS experience the disabling effects of fatigue, point to the importance of alleviating fatigue symptoms. Treatment for fatigue is currently restricted to exercise therapy, energy conservation strategies, cooling therapies, cognitive behavior therapy, mindfulness, and pharmacological interventions including antidepressant therapy and wake-promoting agents. While there is little evidence to suggest a benefit for pharmacological strategies[50], efficacy studies have demonstrated positive effects for cognitive behavior therapy[51], education[50], mindfulness[52], exercise[53,54], and programs involving energy conservation strategies[55] either alone or in combination with a cognitive behavioral approach[56]A recent meta-analysis revealed a significant benefit of exercise and education over pharmacological interventions (modafinil)[50]. Further investigation is required, however, regarding the type of exercise that may be of greatest benefit, and whether certain individuals would experience greater benefit over others.

While there are several recognized important treatments for fatigue in PwMS, our comprehensive exploration of the potential role of lifestyle factors in modifying fatigue from a large international sample of PwMS provides an important addition to the literature. Results of our study demonstrate that there is a range of modifiable lifestyle factors that may minimise or exacerbate fatigue. While numerous sociodemographic and disease factors have been inconsistently linked with severe fatigue (e.g., disease type, employment, disability, comorbidities) [41,57–59], and were confirmed by the present study, our results demonstrated that even after controlling for a broad range of these stable demographic and disease-related factors, fatigue levels can be significantly affected by modifiable factors within the control of PwMS. Exercise, moderate alcohol use, frequent fish consumption, vitamin D supplementation, supplementation with flaxseed oil, healthy diet, normal BMI, and never using a DMD all reduced the odds of having clinically significant fatigue. We have previously demonstrated that moderate alcohol consumption is associated with improved HRQOL and reduced levels of disability (adjusted for age and gender)[60], and hypothesise that the association with reduced levels of fatigue may be, in part, mediated by immunological changes. Regardless of mechanism, these findings should be of value to both clinicians and patients alike when considering factors that may be contributing to an individual’s fatigue, identifying factors amenable to modification, and developing a comprehensive secondary and tertiary preventive approach to MS-related fatigue. Relying solely on DMDs may not be the most effective treatment option for PwMS, given our demonstration of their association with this particularly disabling symptom, and the potential for a more comprehensive preventive strategy utilizing modification of lifestyle risk factors.

### Strengths and Limitations

This study was strengthened by our large, geographically diverse sample which included people with all types of MS and a broad spectrum of levels of disability. While this may improve the generalizability of our sample internationally, generalizability may also be limited by the atypical nature of our sample that is likely to have been affected by the use of web-based recruitment; participants were predominantly female, from English speaking countries, with relatively high levels of education, and are likely to have been highly pro-active about engaging in lifestyle management and self-help, given their use of website used in recruitment.

It is possible that our data may have been affected by responder bias. While data were de-identified upon analysis, survey participation did not permit anonymity. Thus it is possible participant responses were biased towards reporting more healthy lifestyle behavior, and/or a more favorable outcome in terms of morbidity.

All data collected for this study were self-reported. There is, therefore, a possibility of measurement error due to self-report which may bias results through dependent misclassification, where measurement error is correlated between the exposures and outcomes. While the degree to which this is a problem in the present study is unknown, others have found a very high concordance (98.7%) between self-reported MS diagnosis and actual diagnosis[61], and good reliability in other self-reported health outcomes[62,63]. The congruence of our results with those of others reporting on fatigue in MS also suggests a high level of robustness in fatigue data collected.

It is conceivable that some of the findings of this study could be attributed to reverse causality. People with MS who experience high levels of fatigue may refrain from certain activities which may in turn compound the experience of fatigue, while those with low levels of fatigue or disease may be more likely to persist with healthy lifestyle behaviour. Issues of causation may be clarified in planned longitudinal studies of this sample.

Although we used a validated measure of fatigue, our interpretation of data may be constrained by the lack of assessment of potential confounders such as sleep habits, heat sensitivity and cognitive dysfunction; all have been linked to increased levels of fatigue[21,64,65]. Although we explored the impact of DMDs on fatigue, this study did not investigate the association between clinically significant fatigue and the use of pharmacological and non-pharmacological treatments for fatigue. Our study is, however, strengthened by the fact that we controlled for depression risk, and a broad range of disease-related factors including disability.

## Conclusion

There is a wide range of modifiable lifestyle factors that may reduce or contribute to clinically significant fatigue. This study supports strong clinically and statistically significant associations between fatigue in PwMS and the modifiable factors of diet, exercise, omega 3 supplementation, (particularly flaxseed oil), fish consumption, vitamin D supplementation, BMI, alcohol intake, and DMD use. These factors should be considered when devising a comprehensive secondary and tertiary preventive medical approach to managing MS-related fatigue. While caution should be exercised with some associations that may have been contributed to by reverse causality (where health status is the cause rather than the effect), the issue of causation should be clarified in planned longitudinal studies for this sample.
